# Growth Failure and Non-thyroidal Illness in an Infant

**DOI:** 10.7759/cureus.102664

**Published:** 2026-01-30

**Authors:** Shunsuke Shimazaki, Miu Kishimura, Junichi Sato

**Affiliations:** 1 Department of Paediatrics, Funabashi Municipal Medical Center, Funabashi, JPN

**Keywords:** failure to thrive, growth failure, infant, malnutrition, non-thyroidal illness

## Abstract

Reports incorporating pituitary function testing in infants with non-thyroidal illness (NTI) are rare. Here, we describe the case of an infant (a 1-year-3-month-old boy) with failure to thrive secondary to inappropriate feeding practices. The infant developed NTI and reduced IGF-1 levels. He exhibited markedly impaired growth velocity, with low free triiodothyronine (T3), free thyroxine (T4), and insulin-like growth factor 1 (IGF-1) levels despite normal thyroid-stimulating hormone (TSH) levels and intact anterior pituitary responses on stimulation testing.

Neuroimaging revealed no structural abnormalities. Upon nutritional rehabilitation with age-appropriate caloric intake, he exhibited catch-up growth and normalization of thyroid function and IGF-1 levels. This case demonstrates that NTI and low IGF-1 levels may occur in infants with malnutrition. The pathophysiology of NTI involves inflammatory cytokine-mediated alterations, suppression of hypothalamic-pituitary-thyroid axis regulation, and hypoleptinemia during starvation. Low IGF-1 levels reflect growth hormone (GH) resistance, mediated by fibroblast growth factor 21, and reduced insulin and leptin signaling. This case highlights the necessity of evaluating the nutritional and clinical background when assessing endocrine abnormalities to avoid misdiagnosing intrinsic pituitary or thyroid disease. Prompt nutritional intervention is critical for restoring growth and endocrine homeostasis. This case provides insights into the interrelationship between malnutrition, thyroid function, and growth regulation during early childhood.

## Introduction

Non-thyroidal illness (NTI) is characterized by a decline in thyroid hormone levels under conditions of malnutrition or various diseases, despite the absence of intrinsic thyroid abnormalities [1]. Patients with NTI exhibit low serum triiodothyronine (T3) levels without a compensatory increase in thyroid-stimulating hormone (TSH) levels; in some cases, TSH may even be suppressed.

Growth hormone (GH) exerts its effects via the GH receptor, thereby promoting the production of insulin-like growth factor 1 (IGF-1) in the liver. Hepatic GH-dependent IGF-1 synthesis is finely regulated through a complex interplay of nutritional status, hormones, and various growth factors. GH resistance observed under conditions of malnutrition is regarded as an adaptive mechanism that enhances survival during periods of starvation and nutrient deprivation [2].

We encountered the case of an infant who developed NTI and reduced IGF-1 levels secondary to failure to thrive due to the discontinuation of formula and insufficient complementary feeding.

## Case presentation

A 1-year-3-month-old boy was referred to our hospital for poor weight gain. He was born at term via vaginal delivery without asphyxia, with a birth weight of 3620 g, and newborn screening revealed no abnormalities. His developmental milestones were appropriate, with head control at 3 months, rolling over at 6 months, and pulling to stand at 8 months. His past medical history was notable only for cow’s milk allergy, limited to unheated milk. At approximately 10 months of age, he began vomiting after formula intake, and a local pediatrician diagnosed cow’s milk allergy. Subsequently, his mother independently discontinued formula feeding, and he was fed only solid foods at the completion stage, taking three meals daily. On presentation, his height was 70.5 cm (-2.74 SD) and his weight was 7.45 kg (-2.86 SD), with markedly reduced growth velocity (-4.23 SD). He appeared alert and active, with no pallor, goiter, cardiac murmur, respiratory abnormalities, abdominal mass, peripheral coldness, or edema. No relevant family history of thyroid disease was reported.

Laboratory findings showed no abnormalities in hepatic, renal, or electrolyte parameters, and lipid levels were within the normal range (Table 1).

**Table 1:** Laboratory test data. ACTH: Adrenocorticotropic hormone; BE: Base excess; HCO₃: Bicarbonate; IGF-1: Insulin-like growth factor 1; TSH: Thyroid-stimulating hormone; T3: Triiodothyronine; T4: Thyroxine.

Test	At first visit	3 months later	Reference value
Peripheral blood test			
WBC (/µL)	9400	14700	3300-8600
RBC (×10⁴/µL)	343	377	435-555
Hemoglobin (g/dL)	9.7	10.3	13.7-16.8
Hematocrit (%)	29.5	31	40.7-50.1
Platelets (×10⁴/µL)	31.4	39.9	15.8-34.8
Serum biochemical test			
Total protein (g/dL)	7.3	6.8	6.6-8.1
Albumin (g/dL)	4.7	4.1	3.9-5.1
Creatine kinase (U/L)	77	192	59-248
Sodium (mmol/L)	139	139	135-145
Chloride (mmol/L)	106	106	101-108
Potassium (mmol/L)	4.3	4.3	3.6-4.8
Calcium (mg/dL)	9.8	9.7	8.8-10.1
Phosphorus (mg/dL)	4.7	5.7	2.7-4.6
Triglycerides (mg/dL)	124	251	40-149
Total cholesterol (mg/dL)	150	121	142-248
CRP (mg/dL)	<0.05	0.09	0.00-0.14
Venous blood gas test			
pH	7.442	N/A	7.35-7.45
pCO₂ (mmHg)	33.8	N/A	35-45
HCO₃⁻ (mmol/L)	22.5	N/A	21-26
BE (mmol/L)	-1.1	N/A	-2 to +2
Endocrinological test			
TSH (µIU/mL)	1.357	2.281	0.350-4.940
Free T3 (pg/mL)	2.14	3.95	1.68-3.67
Free T4 (ng/dL)	0.76	1.2	0.70-1.48
IGF-1 (ng/mL)	16	64	14-148
ACTH (pg/mL)	49.6	N/A	7.2-63.3
Cortisol (µg/dL)	10.5	N/A	7.07-19.6

**Figure 1:**
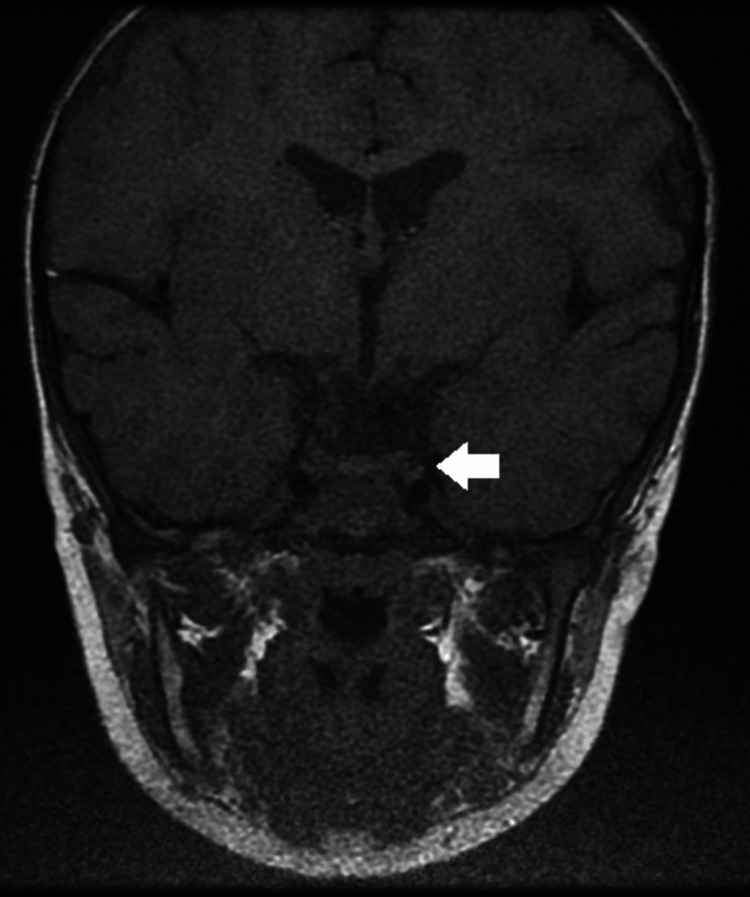
Brain MRI of the pituitary gland. Brain MRI demonstrated no evidence of pituitary hypoplasia or other structural abnormalities.

Endocrine testing revealed decreased free T3, T4, and IGF-1 levels, while TSH remained within the normal range. Brain MRI revealed no evidence of pituitary hypoplasia or other structural abnormalities (Figure 1).

Review of the growth chart indicated a marked decline in weight after 10 months of age, accompanied by slowed linear growth. Insulin tolerance, thyrotropin-releasing hormone (TRH), and luteinizing hormone-releasing hormone (LH-RH) loading tests demonstrated normal GH and cortisol responses, as well as normal TSH dynamics, suggesting intact pituitary function (Table 2).

**Table 2:** Pituitary function test. ACTH: Adrenocorticotropic hormone; FSH: Follicle-stimulating hormone; GH: Growth hormone; IGF-1: Insulin-like growth factor 1; LH: Luteinizing hormone; N/A: Not applicable; TSH: Thyroid-stimulating hormone; T3: Triiodothyronine; T4: Thyroxine.

Time (min)	0	15	30	60	90	120	Reference value
Glucose (mg/dL)	85	43	42	49	56	86	70-109
GH (ng/mL)	4.42	N/A	3.07	8.52	6.75	3.66	>6
ACTH (pg/mL)	26.5	N/A	80.8	34.5	27.8	87.9	28-130.6
Cortisol (µg/dL)	10.7	N/A	17.6	19.2	19.9	23	10.6-26.9
TSH (µIU/mL)	1.504	N/A	1.504	10.809	9.044	7.081	3.6-26.8
Free T3 (pg/mL)	2.68	N/A	N/A	N/A	N/A	3.06	1.68-3.67
Free T4 (ng/dL)	0.84	N/A	N/A	N/A	N/A	0.84	0.70-1.48
Prolactin (ng/mL)	16.8	N/A	16.8	42.4	33.5	29	4.29-70
LH (mIU/mL)	<0.10	N/A	1	0.9	0.8	0.7	0-6.0
FSH (mIU/mL)	0.4	N/A	3.2	4.3	4.4	4.5	0-15.6

Because caloric insufficiency due to discontinuation of formula milk was suspected, caregivers were instructed to provide a nutritionally adequate diet of approximately 1000 kcal/day (130 kcal/kg/day), appropriate for the patient’s age.

**Figure 2:**
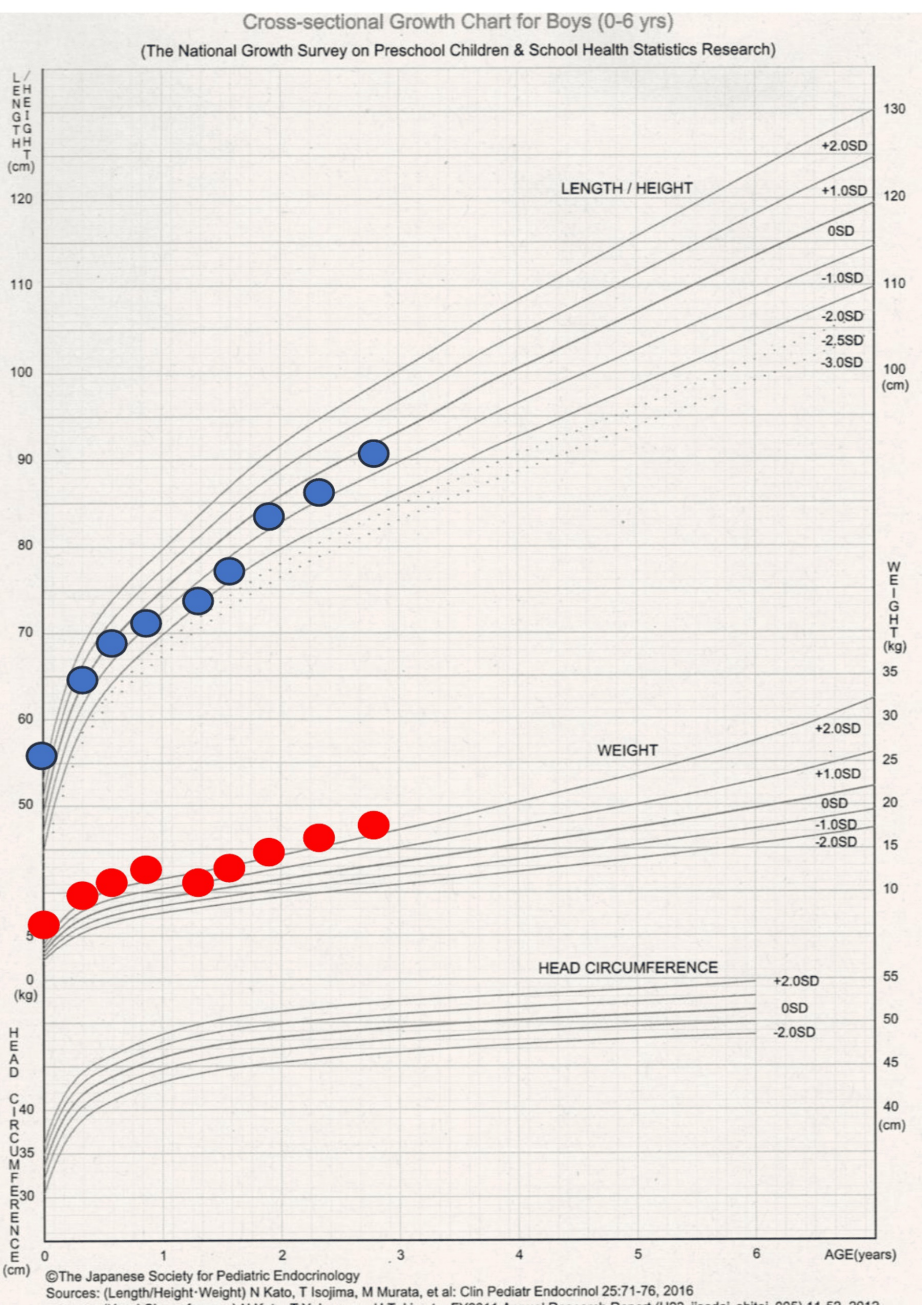
Growth curve of the patient. Source: Reference [3].

Following this intervention, both height and weight improved, with catch-up growth documented three months later, along with normalization of thyroid function tests and IGF-1 levels (Table 1, Figure 2).

These findings indicated that thyroid dysfunction reflected NTI and that low IGF-1 levels were secondary to poor weight gain. Continuous outpatient follow-up confirmed favorable growth and developmental progress.

## Discussion

Reports documenting pituitary function testing in infants with NTI, as illustrated in this case, are scarce. Thyroid hormone levels decrease in response to starvation and systemic illnesses. Initially, serum T3 levels decline, and with increasing severity and duration, T4 levels decrease. During this process, TSH levels may fail to increase and may even be suppressed. Previously termed “low T3 syndrome” or “euthyroid sick syndrome (ESS),” the condition is now referred to as NTI [1].

NTI can arise in diverse conditions, including malnutrition (e.g., starvation and anorexia nervosa), sepsis, diabetes mellitus, liver cirrhosis, renal failure, malignancy, and systemic diseases [4]. It may also occur after surgery, trauma, or administration of drugs such as corticosteroids and β-blockers.

The mechanisms underlying NTI are multifactorial. One such factor is the production of inflammatory cytokines. Boelen A et al. reported a negative correlation between IL-6 and serum T3 levels [5]. In IL-6 knockout mice, the decline in serum T3 levels during acute illness was attenuated [6]. Other inflammatory cytokines, including IL-1β, soluble IL-2 receptor, and TNF-α, have also been shown to be elevated in NTI associated with sepsis [7]. Recent molecular studies have revealed that inflammatory cytokines influence thyroid hormone metabolism and contribute to the pathophysiology of NTI.

Another factor is alteration of the hypothalamic-pituitary-thyroid axis set point. Suppression of thyrotropin-releasing hormone gene expression in the paraventricular nucleus prevents TSH elevation despite reduced T3 levels [8]. Leptin, an adipocyte-derived hormone, decreases during starvation, and hypoleptinemia further suppresses thyrotropin-releasing hormone expression [9]. Thus, changes in central regulation play a key role.

In our case, low serum IGF-1 levels and reduced growth velocity prompted anterior pituitary testing. IGF-1, which is produced by hepatocytes and chondrocytes in response to GH stimulation, promotes cell growth by inducing chondrocyte differentiation and proliferation. IGF-1 levels are sensitive to nutritional status; GH secretion increases during malnutrition, but IGF-1 levels decrease because of GH resistance. Thus, IGF-1 serves as a sensitive marker of acute nutritional disturbance. Although our patient did not show clear evidence of increased GH secretion, the rapid recovery of IGF-1 following nutritional improvement suggested that the findings were attributable to malnutrition.

The mechanism underlying GH resistance involves fibroblast growth factor 21, which is upregulated in the liver during malnutrition [10]. Fibroblast growth factor 21 induces peroxisome proliferator-activated receptor γ coactivator 1-α expression, promoting β-oxidation and gluconeogenesis. It simultaneously inhibits GH-dependent signal transducer and activator of transcription 5 phosphorylation in the liver, thereby leading to GH resistance. Additionally, decreased insulin secretion during starvation suppresses hepatic GH receptor expression, and hypoleptinemia alters the central regulation of GH secretion.

Similar cases of NTI in infants have been reported [11]. For example, Sato T et al. described the case of an infant with transient NTI in whom thyroid function normalized in parallel with catch-up growth, suggesting that NTI may significantly affect infant development. In our case, no psychomotor developmental delay was observed at the time of diagnosis, and subsequent developmental milestones progressed favorably.

## Conclusions

This case demonstrates that NTI and low IGF-1 levels may occur not only in adults or older children but also in infants with malnutrition. In infants, prolonged NTI is of particular concern because of its potential adverse effects on growth and neurodevelopment and therefore warrants careful clinical attention. When endocrine abnormalities are encountered, the patient’s nutritional and clinical background must be carefully evaluated.
